# Analysis of Proteins and Peptides of Highly Purified CD9^+^ and CD63^+^ Horse Milk Exosomes Isolated by Affinity Chromatography

**DOI:** 10.3390/ijms232416106

**Published:** 2022-12-17

**Authors:** Sergey E. Sedykh, Lada V. Purvinsh, Evgeniya E. Burkova, Pavel S. Dmitrenok, Elena I. Ryabchikova, Georgy A. Nevinsky

**Affiliations:** 1 SB RAS Institute of Chemical Biology and Fundamental Medicine, Lavrentieva Ave. 8, Novosibirsk 630090, Russia; 2 Faculty of Natural Sciences, Novosibirsk State University, Pirogova 1, Novosibirsk 630090, Russia; 3 Institute of Biomedical Systems and Biotechnologies, Peter the Great St. Petersburg Polytechnic University, Polytechnicheskaya, 29, St. Petersburg 195251, Russia; 4 Elyakov Pacific Institute of Bioorganic Chemistry of FEB RAS, 100 let Vladivostoku Ave. 159, Vladivostok 690022, Russia

**Keywords:** horse milk, extracellular vesicles, exosomes, peptides, proteins, mass spectrometry, tetraspanins, CD9, CD63

## Abstract

Exosomes are nanovesicles with a 40–150 nm diameter and are essential for communication between cells. Literature data suggest that exosomes obtained from different sources (cell cultures, blood plasma, urea, saliva, tears, spinal fluid, milk) using a series of centrifugations and ultracentrifugations contain hundreds and thousands of different protein and nucleic acid molecules. However, most of these proteins are not an intrinsic part of exosomes; instead, they co-isolate with exosomes. Using consecutive ultracentrifugation, gel filtration, and affinity chromatography on anti-CD9- and anti-CD63-Sepharoses, we isolated highly purified vesicle preparations from 18 horse milk samples. Gel filtration of the initial preparations allowed us to remove co-isolating proteins and their complexes and to obtain highly purified vesicles morphologically corresponding to exosomes. Using affinity chromatography on anti-CD9- and anti-CD63-Sepharoses, we obtained extra-purified CD9^+^ and CD63^+^ exosomes, which simultaneously contain these two tetraspanins, while the CD81 tetraspanin was presented in a minor quantity. SDS-PAGE and MALDI analysis detected several major proteins with molecular masses over 10 kDa: CD9, CD63, CD81, lactadherin, actin, butyrophilin, lactoferrin, and xanthine dehydrogenase. Analysis of extracts by trifluoroacetic acid revealed dozens of peptides with molecular masses in the range of 0.8 to 8.5 kDa. Data on the uneven distribution of tetraspanins on the surface of horse milk exosomes and the presence of peptides open new questions about the biogenesis of these extracellular vesicles.

## 1. Introduction

Milk is a source of hundreds of unique proteins; thousands of publications devoted to milk proteins have been indexed in PubMed in the past ten years. Milk contains many different biologically active proteins [1] and peptides [2]: lactoferrin, antibodies [3], caseins, and many others, which are essential for the newborn. Other biologically active components of milk are mono- and oligosaccharides [1], lipids [4], nucleotides [5] and nucleic acids [6], vitamins [7], lipids [7], etc. Natural milk compounds contribute to the newborn’s growth and protect a child from various infections. These molecules are usually considered integral parts of infants’ intestinal physiology [8,9].

Extracellular vesicles are presented with microparticles, microvesicles, exosomes, apoptotic bodies, ectosomes, and other subsets of extracellular vesicles [10]. Exosomes are vesicles of particular interest since they are a unique source of proteins and nucleic acids with a high diagnostic potential [11,12,13]. Exosomes provide a versatile and efficient way for the targeted delivery of drugs [14,15] into cells. The advantages of exosomes over other delivery methods are their low cytotoxicity, size, low immunogenicity, and expression of specific surface markers [16]. Milk exosomes are of particular interest because bovine [17] and human [18] milk exosomes successfully enter cells.

Various mechanisms have been proposed for the delivery of biologically active substances using exosomes, both inside the vesicles and on the surface, for example, by functionalizing the surface of exosomes with peptides [19], transfection with commercial reagents, ultrasound, electroporation [20], liposomes [21], as well as cell and genetic engineering of cell cultures from which exosomes are obtained [22].

Exosomes are usually isolated from the biological liquids by centrifugations and ultracentrifugations [23,24,25]. However, all these conventional approaches result in preparations containing exosomes and many co-isolating components, including proteins, nucleic acids, and different complexes. Exosome preparations isolated from bovine milk after centrifugation and ultracentrifugation in the sucrose gradient contained 2107 proteins [9], including typical exosome markers, whose number is highly overestimated.

Biological fluids contain many proteins and complexes, which can be co-precipitated with vesicles during ultracentrifugation. For example, the human placenta and milk contain a stable high molecular mass (~1 MDa) multiprotein complex with a size comparable to extracellular vesicles [26,27]. Some proteins or protein complexes are associated with the vesicle surface or the receptors. We suggest that the number of proteins in crude exosome preparations obtained by centrifugations and ultracentrifugations may be significantly overestimated [28,29,30].

Preparations of exosomes isolated with ultracentrifugation may contain various structures of low electron density without membranes named “non-vesicles” [31]. Transmission electron microscopy revealed two types of “non-vesicles”: 20–40 nm diameter, corresponding to 10–40% of all particles, and 40–100 nm diameter. The morphology of “non-vesicles” allows attributing them to lipoproteins of low and intermediate density (20–40 nm) or very low density (40–100 nm). Such preparations isolated from the biological fluids by centrifugations and ultracentrifugations may contain dozens and thousands of co-precipitating proteins.

Exosomes were isolated from human milk for the first time in 2007 [23]; since then, they have been obtained in bovine, porcine, wallaby, camel, rat, horse, panda, yak, sheep, and goat milk [30]. Most of the papers describe the proteins of milk exosomes isolated using centrifugations and ultracentrifugations. Such crude preparations contain up to thousands of individual proteins [32] and peptides, microRNA [33], and mRNA [34]. Since no cases of prion diseases (e.g., bovine spongiform encephalopathy) were described in horses and, accordingly, the transmission of prions through horse milk is excluded, exosomes isolated from the horse milk are of particular interest for possible use in drug delivery and therapy.

As shown for the horse milk exosomes, the subfraction of co-isolating proteins may be easily separated from the vesicles using gel filtration [29,35]. In 2017, we described a number of major proteins (CD9, CD81, CD63, actin, lactadherin, lactoferrin, butyrophilin, and xanthine dehydrogenase) in the preparations of horse milk exosomes after gel filtration [35]; later, in 2021, we identified 29 peptides of 0.8–8.8 kDa after affinity chromatography on anti-CD81-Sepharose [29]. We obtained the same results on the number of proteins and peptides in the preparations of human placenta exosomes after gel filtration (13 proteins) [36] and chromatography on anti-CD81-Sepharose (27 peptides) [37]. The critical review of academician Evgeny Sverdlov [38] supports our view that the number of biological components in exosomes is overestimated in scientific papers. Another concern is whether hundreds and thousands of proteins inside (and outside) milk exosomes have any physiological significance.

Here, we show the results of the analysis of horse milk exosomes affinity-purified on anti-CD9/CD63-Sepharose, containing immobilized polyclonal rabbit IgG against corresponding tetraspanins. Since the affinity-purified CD9+ and CD63+ exosomes contain traces of CD81 tetraspanin, our results are in favor of the uneven distribution of tetraspanins on the exosomal surface and pose a question of whether this is a peculiar specificity of horse milk exosomes or a general rule. We suggest that further biochemical analysis of highly purified horse milk exosomes is essential for their use as drug delivery vehicles.

## 2. Results

### 2.1. Purification of Exosomes

**Figure 1:**
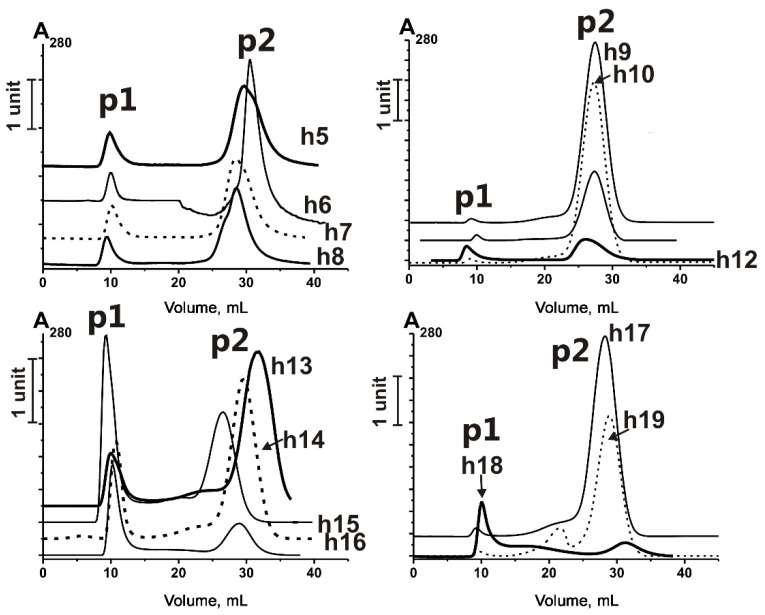
Profiles of Ultrogel A4 gel filtration of horse milk exosome-enriched fractions obtained using ultracentrifugation. p1—the first peak of elution, containing exosomes, p2—the second elution peak, containing co-precipitated impurities. h5–h19—horse milk samples, used for the exosome isolation (for data on h1–h4, see [35]).

Since the crude exosome preparations isolated from different biological fluids using centrifugations and ultracentrifugations contain hundreds to thousands of different proteins, the additional steps of exosome-enriched fraction purification are needed [28,31,39]. Our recent studies show that further purification of horse milk exosomes obtained using ultracentrifugation by gel filtration removes most co-precipitating protein impurities [29,35]. Figure 1 demonstrates typical gel filtration profiles of horse milk exosome-enriched preparations isolated from 14 milk samples.

As shown in Figure 1, peak p1 is distinctly separated from the p2 peak of co-isolating proteins and protein complexes. As we have shown in [35], p2 contains various proteins described in milk exosome preparations obtained by centrifugations and ultracentrifugations [39] and includes “negative exosome markers” such as casein and other proteins [30]. The p1 contains only several major proteins: CD9, CD63, CD81, lactadherin, actin, butyrophilin, lactoferrin, and xanthine dehydrogenase. We used aliquots of p1 from 14 horse milk exosome preparations obtained by gel filtration (VESmix) and identified the proteins using SDS-PAGE and trypsin hydrolysates [35] and identified the same major proteins.

**Figure 2:**
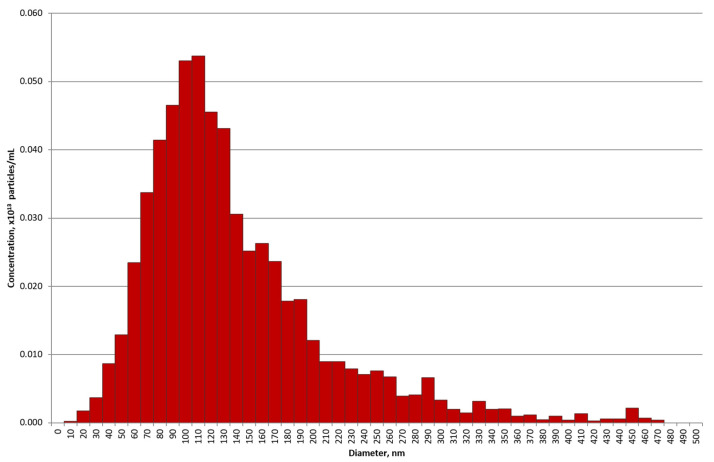
Nanoparticle tracking analysis of horse milk exosome preparation obtained by gel filtration of sediment after ultracentrifugation. The profile of exosome size distribution is presented.

According to the nanoparticle tracking analysis (NTA) results, the preparations of horse milk vesicles after gel filtration are represented mainly by particles with a diameter of 70–140 nm (Figure 2), corresponding in size to exosomes. The aliquot of the preparation, corresponding to the number of exosomes isolated from about 50 mL of milk, according to the NTA contains 0.61 × 10^13^ particles/mL (the number of particles in the buffer solution containing no exosomes did not exceed 10^9^ particles/mL).

The preparations of horse milk exosomes obtained with gel filtration were further separated on anti-CD9- and anti-CD63-Sepharose.

### 2.2. Affinity Chromatography on Anti-CD9/CD63-Sepharose

**Figure 3:**
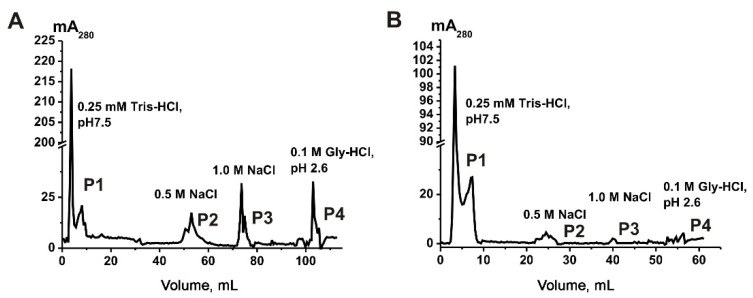
Affinity chromatography of VESmix on anti-CD9- (**A**) and anti-CD63-Sepharose (**B**). (**–**)—absorbance on A_280_ nm, mAU; P1—subfraction appeared in flow, P2—subfraction eluted with 0.5 NaCl, P3—subfraction eluted with 1.0 M NaCl, P4—subfraction eluted with 0.1 M Gly-HCl pH 2.6.

According to the MISEV-2018 [10], exosomes contain CD9, CD63, and CD81 tetraspanins in their membranes. Using affinity chromatography with anti-CD81-Sepharose, we described the subfraction of horse milk exosomes containing CD81 in their membranes [29]. We used the same methodology—affinity chromatography of VESmix—on anti-CD9- (Figure 3A) and anti-CD63-Sepharose (Figure 3B). As shown in Figure 3, a significant subfraction of vesicles appears in flow (see peak P1 corresponding to the elution with 0.25 M Tris-HCl). We can interpret P1 as subfraction of exosomes containing no CD9 or CD63 tetraspanins or with a low density of these tetraspanins in the exosomal membrane. Exosomes showing affinity to anti-CD9/CD63-Sepharose were eluted from the corresponding columns in three peaks by buffer solution containing 20 mM Tris HCl pH 7.5 and 0.5 M NaCl (P2), 1.0 M NaCl (P3), and 0.1 M Gly-HCl pH 2.6 (P4). Interestingly, P2–P4 corresponding to the anti-CD63-Sepharose elution (Figure 3B) are significantly smaller than in the case of anti-CD9-Sepharose (Figure 3A).

The elution of protein compounds and protein-containing supramolecular complexes such as exosomes from affinity sorbents containing immobilized antibodies with various salt concentrations and/or acidic buffer is a general approach [40]. Our previously published papers confirm the stability of exosomal shape, structure, and surface markers under the described conditions.

### 2.3. Electron Microscopy Analysis

Exosomes isolated using centrifugations and ultracentrifugations contain vesicles of different sizes, non-vesicles, exosomes, proteins, and protein complexes [29,31,36]. We have shown that exosomes isolated from human placenta homogenates must be purified by gel filtration [36,37] to be pure enough for proteomic analysis [31,41,42].

**Figure 4:**
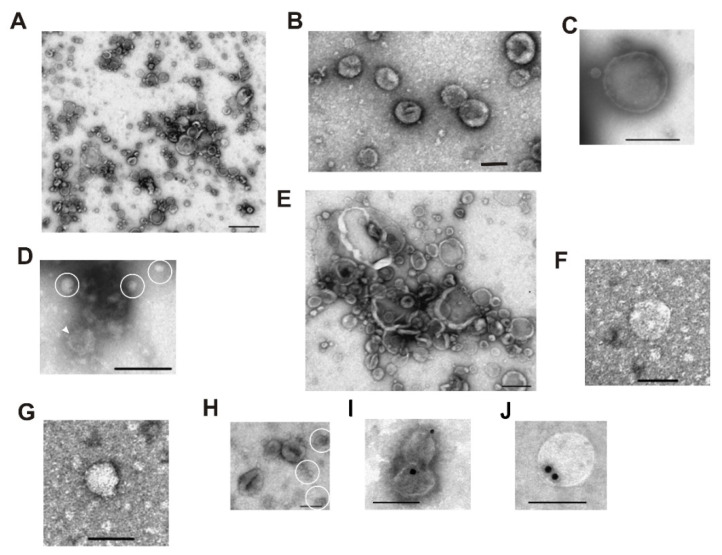
Transmission electron microscopy of horse milk exosomes on different stages of isolation and purification. P2 subfraction was obtained after affinity chromatography on anti-CD9-Sehparose (**A**–**D**,**I**) and anti-CD63-Sepharose (**E**–**H**,**J**). General view (**A**,**E**); exosomes of ≤ 100 nm (**B**,**F**); vesicles > 100 nm (**C**); exosomes (white arrow) and particles of medium electron density without outer membranes (ovals) (**D**); particles of medium electron density without outer membranes (**G**); vesicles, “non-vesicles”, particles of medium electron density without outer membranes (**H**); gold immunolabeling using anti-CD9-IgG (**I**) and anti-CD63-IgG (**J**). The length of the scale line corresponds to 100 nm (**B**,**D**,**F**–**J**) and 200 nm (**A**,**C**,**E**).

The transmission electron microscopy results shown in Figure 4 confirm that gel filtration and affinity chromatography on anti-CD9/CD63-Sepharose allow obtaining exosome preparations that do not contain undesirable contaminations as any proteins and their complexes (Figure 4A,B).

Transmission electron microscopy of subfractions of VESmix preparation separated by affinity chromatography on anti-CD9/CD63-Sepharose has shown that P2 subfraction eluted with 0.5 M NaCl contained exosomes, vesicles, and their associates of different sizes. Proteins and their associates were absent in these subfractions. However, even affinity chromatography does not remove the vesicles >100 nm (Figure 4C) completely and particles of medium electron density without outer membranes (Figure 4D,G). Compared to the P2 and P3 subfractions, P1 and P4 contain an increased concentration of “non-vesicles” and their associates (data not shown); P4 contains particles of very low, low, and medium electron density without outer membranes and vesicles >100 nm. Thus, we used P2 + P3 for further analysis. Gold immune labeling using protein A-gold conjugate and anti-CD9-IgG and anti-CD63-IgG (Figure 4I,J) confirmed the exosomal nature of vesicles, eluted from anti-CD9/CD63-Sepharose.

### 2.4. Flow Cytometry Analysis

**Figure 5:**
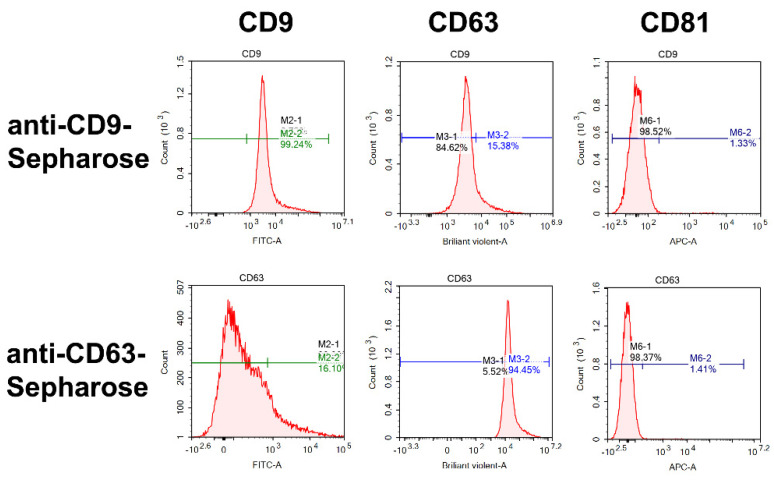
Flow cytometry analysis of P2 + P3 subfractions eluted from anti-CD9- and anti-CD63-Sepharose. Relative amounts of exosomes containing CD9 (left panel), CD63 (central panel), and CD81 (right panel) are shown.

Using the protocols we described earlier for horse milk and human placenta exosomes [29,37], the relative amount of horse milk exosomes eluted from anti-CD9/CD63-Sepharose (P2 + P3) containing CD9, CD63, and CD81 tetraspanins was estimated using flow cytometry. The P2 + P3 fractions were selected for analysis due to the transmission electron microscopy data (shown in Section 2.3), according to which P2 and P3 subfractions contained most of the exosomes. As one can see in Figure 5, exosomes eluted from anti-CD9-Sepharose contain on their surface CD9 (99.2%) and CD63 (15.4%), while the CD81 tetraspanin is underrepresented (1.3%). Reciprocally, the P2 + P3 subfractions of horse milk exosomes eluted from anti-CD63-Sepharose contained CD9 (16.1%) and CD63 (94.45%), and traces of CD81 (1.1%). Thus, we suggest that CD9 and CD63 tetraspanins are co-presented on horse milk exosomes, which is not true for the CD81 tetraspanin.

Our results of exosome purification on anti-CD9- and anti-CD63-Sepharose are consistent with the results we obtained on anti-CD81-Sepharose in [29]: CD9—16.5%, CD63—6.1%, CD81—74.8%. These results testify that CD9 is the most represented tetraspanin (83.9% of exosomes appeared in flow during anti-CD81-Sepharose chromatography contained CD9). We also confirm our results suggesting that exosomes may simultaneously contain at least two tetraspanins—CD9 and CD63—and other combinations in much smaller proportions: CD63 and CD81, CD9 and CD81, CD63 and CD81. Our results explain why we did not obtain a significant amount of exosomes after sequential affinity chromatography: for example, application of P2 + P3 subfraction eluted from anti-CD9-Sepharose to anti-CD63-Sepharose, leading to elution of most exosomes in P1 (appeared in flow). Nevertheless, according to the papers devoted to the milk exosomes (as well as isolated from any other sources) published to the current time, we show here the simultaneous existence of CD9 and CD63 in the significant subfraction of horse milk exosomes for the first time. Taking into account the data on NTA of exosomes, there is no doubt that the extracellular vesicles we obtained are exosomes in terms of size, morphology, and the presence of surface markers according to the MISEV-2018 [10].

### 2.5. Analysis of Peptides and Proteins

In our previous papers, we analyzed proteins and peptides contained in highly purified exosomes from human placenta and horse milk isolated using ultracentrifugations, gel filtration, and affinity chromatography on Sepharose containing immobilized antibodies against CD63 and CD81 tetraspanins [29,35,36,37]. In the case of the horse milk exosome, preparations separated on anti-CD63-Sepharose (P2 + P3) contained the same major proteins revealed after gel filtration: CD9, CD63, CD81, lactadherin, actin, butyrophilin, lactoferrin, and xanthine dehydrogenase [29] (see Table S1). Besides the proteins, exosomes isolated from the human placenta [37] and horse milk [29] also contained peptides with molecular masses lower than 12 kDa. Since it is impossible to detect small proteins and peptides with a molecular mass lower than 10 kDa using standard SDS-PAGE and/or 2D-electrophoresis, the running out of these molecules from the gels during Coomassie staining, we used direct MALDI-TOF analysis. The peptides found in human placenta and horse milk exosomes might arise from different sources, but they are still practically not investigated.

Here, we show that exosomes obtained using gel filtration on Ultrogel A4 and subsequent affinity chromatography on anti-CD9/CD63-Sepharose contain the same proteins after their purification by gel filtration. The main objective was the analysis of peptides.

**Figure 6:**
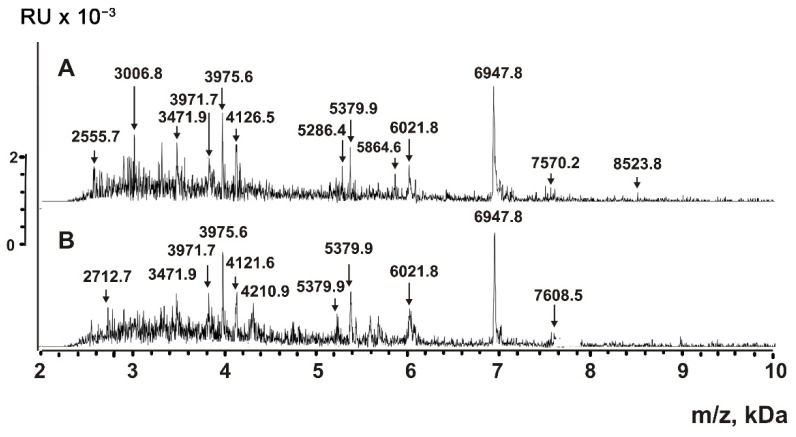
Direct MALDI mass spectrometry analysis of untreated exosomes (P2 + P3) after affinity chromatography on anti-CD9- (**A**) and anti-CD63-Sepharose (**B**). The errors in determining the *m*/*z* value did not exceed 0.5–1.0 Da.

It was shown that small proteins and peptides of bacteria could be detected by MALDI mass spectrometry directly in mixtures of native microorganisms and HCCA (α-Cyano-4-hydroxycinnamic acid) matrix [41,42]. The peptide detection sensitivity is higher than for large proteins with molecular mass >10 kDa. Due to the easier ionization of peptides, peaks of oligonucleotides, lipids, sugars, and other compounds are distinguished at 100–1000-fold lower concentrations. We mixed VESmix preparations from anti-CD9/CD63-Sepharoses with HCCA matrix and applied them on steel plates for MALDI mass spectrometry analysis in 2–12 kDa (Figure 6). The analysis of native exosomes by MALDI mass spectrometry demonstrated that they contain various molecules with molecular mass from 2.5 to 8.5 kDa.

**Figure 7:**
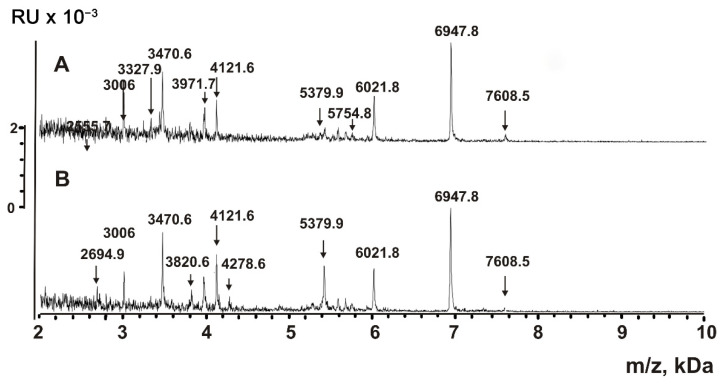
MALDI mass spectrometry analysis of P2 + P3 exosome extracts obtained after affinity chromatography on anti-CD9- (**A**) and anti-CD63-Sepharose (**B**). The extracts were obtained after the destruction with TFA and acetonitrile. The error in determining the *m*/*z* values did not exceed 0.5–1.0 Da.

Aliquots of P2 + P3 fractions of VESmix were processed with TFA to destroy any non-covalent interactions. The insoluble compounds were removed by centrifugation; supernatants were filtered through Amicon centrifuge filters with a 10 kDa cut-off. MALDI mass spectra obtained in the 2–12 kDa range are shown in Figure 7. The spectra contain multiple peaks corresponding to compounds with molecular masses from 2.0 to 7.6 kDa.

**Figure 8:**
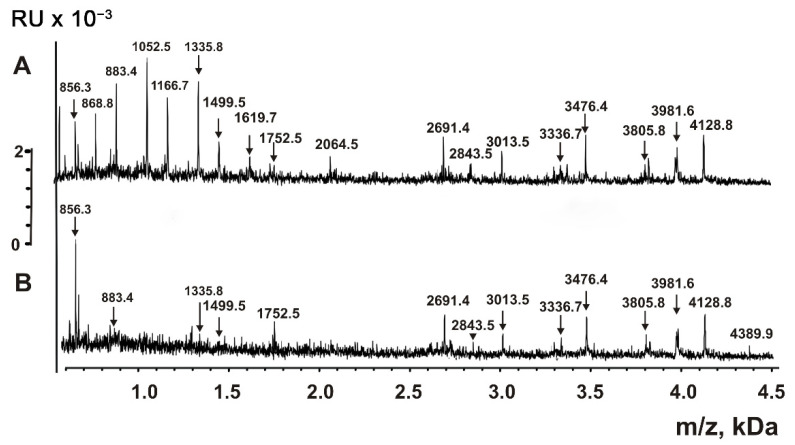
MALDI mass spectra of P2 + P3 subfraction of horse milk exosome extracts obtained after affinity chromatography on anti-CD9- (**A**) and anti-CD63-Sepharose (**B**). The extracts were obtained by destruction of P2 subfraction with TFA and acetonitrile, and the compounds passed through the Amicon filter with a 3 kDa cut-off were analyzed. The error in determining the *m*/*z* values did not exceed 0.5–1.0 Da.

Other aliquots of P2 + P3 fractions of VESmix passed through the Amicon filters with 10 kDa cut-off were additionally filtered through Amicons with 3 kDa cut-off. As shown in Figure 8, the spectra of compounds that passed through the 3 kDa Amicons contain peaks corresponding to compounds with molecular masses from 856 to 4390 Da.

**Figure 9:**
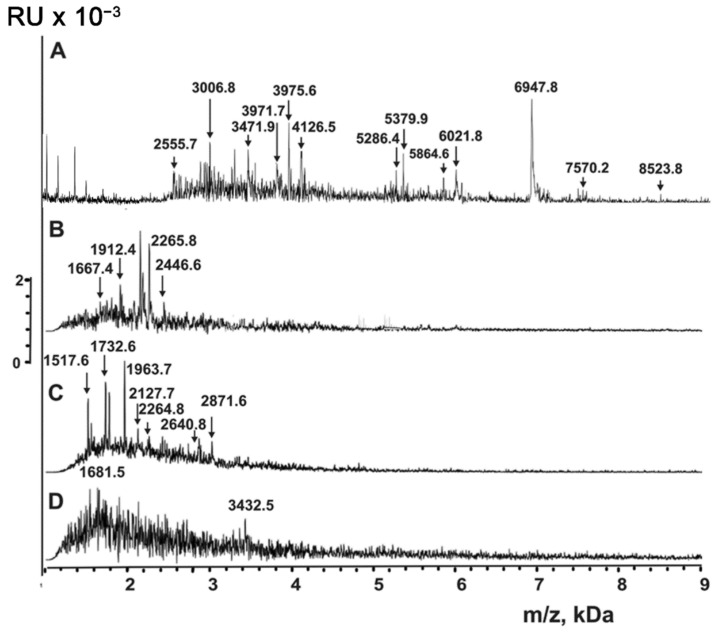
MALDI mass spectra of horse milk exosome extract corresponding to peak P2 after affinity chromatography on anti-CD9-Sepharose before (**A**) and after proteolysis with trypsin (**B**), chymotrypsin (**C**), and proteinase K (**D**). The error in determining the *m*/*z* value did not exceed 0.5–1.0 Da.

As noted above, the HCCA matrix allows the detection of compounds with low molecular masses. We assumed that the molecules contained in P2 + P3 of exosomes after anti-CD9/CD63-Sepharose correspond to peptides of native exosomes. We treated exosome extracts with a molecular mass lower than 10 kDa with trypsin, chymotrypsin, and proteinase K (Figure 9). As one can see, the peaks of the compounds with corresponding molecular masses higher than 3.0 kDa disappeared, and at the same time, hydrolysis products with lower molecular masses appeared. One can notice that the masses of the hydrolysis products do not match those of the initial preparations.

The treatment of P2 + P3 subfraction of horse milk exosomes with TFA can, in principle, lead to the partial acid hydrolysis of proteins with molecular masses higher than 10 kDa, resulting in the formation of peptides. Figure 6, Figure 7, Figure 8 and Figure 9 show that the major peaks corresponding to the peptides are present in the spectra of native vesicles. Therefore, all the peaks detected by MALDI mass spectrometry should be attributed to peptides of untreated native exosomes and their fragments. These peptides were detected in horse milk exosomes containing CD9 and CD63 tetraspanins for the first time.

MALDI TOF mass spectrometry is a semi-quantitative method that only provides information on molecular masses. The corresponding signal will be very high if the particular compound efficiently crystallizes with the MALDI matrix and is easily protonated. Simultaneously, the signals of other compounds might be very low or even completely suppressed. Therefore, it is impossible to obtain the spectra of all short proteins and peptides contained in the mixture. Nevertheless, the direct analysis of untreated exosomes (Figure 6) and their extracts (Figure 7 and Figure 8) indicates that the exosomes contain many peptides from 0.8 to 8.5 kDa. The use of proteases (Figure 9) confirms the peptide nature of corresponding peaks. The major peaks corresponding to the peptides of exosomes containing CD9 and CD63 tetraspanins are presented in all spectra: for example, 3971.7, 5379.9, 6021.8, and 6947.8 (Figure 6, Figure 7, Figure 8 and Figure 9). As one can see, horse milk exosomes contain more than 20 intrinsic peptides.

While we have no doubts about the presence and biological functions on the number of proteins detected by the MALDI in extra-purified horse milk exosomes, we can only make assumptions about the physiological function of peptides, the intrinsic exosomal nature of which we also have no doubts about. It is known that the peptide hormone oxytocin plays an important role in pregnancy and lactation. Opioid peptides formed from caseins are important for the nervous system of a breastfed child [43]. The hypotensive peptides lactorphins, derived from alpha-lactalbumin and beta-lactoglobulin, have been shown to reduce blood pressure in hypertensive rats [44]. The milk of camels, cows, and sheep has also been shown to contain antioxidant peptides, ACE inhibitors, anti-diabetic and anti-obesity peptides, and antimicrobial peptides [45]. The question on the biological function of milk exosome peptides remains to be answered.

## 3. Discussion

We have previously shown that most proteins co-isolating with milk exosomes during ultracentrifugation may be removed by gel filtration [35]. In this paper, we purified horse milk exosomes isolated by gel filtration using affinity chromatography on anti-CD9/CD63-Sepharose. The peaks eluted with 0.5 M (P2) and 1.0 (P3) NaCl from the sorbents contained the maximal number of typical exosomes (40–100 nm) and minima of co-isolating proteins and their complexes. These exosome preparations contained only a few major proteins, previously identified by MALDI mass spectrometry of SDS-PAGE trypsin hydrolysates after gel filtration.

Peptides possess various biological functions: they serve as hormones, neurotransmitters, antibiotics, antioxidants, regulators of growth, blood pressure, and regulate the concentration of calcium, glucose, etc. Previously, we described intrinsic peptides in CD81^+^ horse milk exosomes [29] and human placenta exosomes [37]. Here, we tested two hypotheses: (1) whether horse milk exosomes may simultaneously carry the CD9 and CD63 tetraspanins in their membranes and whether the distribution of tetraspanins is even between exosomes, and (2) if they contain any intrinsic peptides. We used preparations of CD9^+^ and CD63^+^ exosomes, isolated by affinity chromatography on Sepharose bearing immobilized polyclonal rabbit anti-CD9/CD63-IgG. Before and after exosome destruction using TFA, the exosomes contained peptides with molecular masses from 0.8 to 8.6 kDa (Figure 6, Figure 7, Figure 8 and Figure 9). The peptide nature of the compounds was confirmed by protease cleavage (Figure 9). The masses of major peptides corresponding to Figure 6, Figure 7, Figure 8 and Figure 9 are summarized in Table 1.

The data shown in Table 1 point out that most of the exosomal peptides of CD9/CD63-containing horse milk exosomes are common with CD81^+^ horse milk exosomes described in [29]. We do not yet have any data on the specific biological functions of these exosomal peptides, since the amino acid sequences were not determined by MALDI. We hope that high-throughput methods of peptide analysis (LC-MS/MS and others) will allow us to detect the sequences of the peptides of horse milk exosomes. Our further research will be focused on the structure and possible biological significance of the exosomal peptides.

However, as high-throughput methods of peptide sequencing rely on the search of peptides generated after trypsinolysis in databases, we cannot exclude that the peptides we identified are not parts of known proteins or do not contain any sites of trypsin hydrolysis. Thus, even high-throughput methods may be powerless to provide any valuable data.

Some of the differences between CD9^+^, CD63^+^, and CD81^+^ horse milk exosomes may reflect the content of different combinations of protein markers in the surface of the exosomes. In this paper, we have shown uneven distribution of CD9 and CD63 in horse milk exosomes compared to CD81.

## 4. Materials and Methods

### 4.1. Materials

Samples of horse milk from Siberian breed healthy animals were obtained on the Verkh-Irmen’ dairy farm (Novosibirsk region, Russia). The chemicals (Tris, Glycine, Acetic acid, other components of buffers, proteases, components of gels, and reagents for electron microscopy) were obtained from Sigma (St. Louis, MO, USA) and MP Biomedicals (Eschwege, Germany). The horse milk sampling and analysis protocol satisfied the requirements of the bioethical committee protocol no. 201 of the Institute of Chemical Biology and Fundamental Medicine of SB RAS and the recommendations of the European Committee (Council Directive of European Communities 135 86/609/CEE).

### 4.2. Isolation of Exosomes from Horse Milk

The samples of fresh horse milk immediately after milking were refrigerated to +4 °C and were centrifugated at 16,500× *g* using a JA-14 rotor on Avanti J-E centrifuge (Beckman Coulter, Brea, CA, USA) for the removal of lipid fractions, proteins, and cellular debris precipitation. If exosomes were not immediately isolated from milk preparations, they were frozen at −70 °C for several weeks or months without significantly decreasing exosome yield.

The isolation of exosomes and their purification were carried out as in [29,35,39]. The principal (critical) stages of selection were as follows. All the solutions used contained 0.1–1% of Penicillin–Streptomycin–Amphotericin B, 100X solution (MP Biomedicals, Eschwege, Germany) to prevent bacterial growth. The samples up to 1.5 L of horse milk were defrosted from −70 °C and twice centrifugated at 16,500× *g* (JA-14 rotor). Then, 2.4 M sodium acetate was added to 50 mM of the supernatant, and the solution was stirred on a magnetic stirrer, titrated with glacial acetic acid to pH 4.4, and left overnight at +4 °C. The mixture was centrifuged for 20 min at +4 °C at 16,500× *g* (JA-14 rotor), and the precipitate containing milk proteins was removed. The supernatant was titrated with a saturated solution of Tris on a magnetic stirrer until it reached pH 7.5. The solution was centrifuged for 20 min at +4 °C at 16,500× *g* (JA-14 rotor), and the precipitate was removed.

The supernatant was filtered through a TPP 99505 filter with a pore diameter of 0.22 µm (Techno Plastic Products AG, Trasadingen, Switzerland), and ultrafiltrate was additionally centrifuged for 120 min at 16,500× *g* at +4 °C (rotor JA-30.50, centrifuge Avanti J-30I; Beckman Coulter, Brea, CA, USA). The supernatant was ultracentrifuged for 120 min at 100,000× *g* at +4 °C to precipitate the vesicles. We removed the supernatant and resuspended the gelatinous colorless pellet in TBS buffer (20 mM Tris-HCl, pH 7.5, 150 mM NaCl). The preparation was centrifuged for 20 min at 12,000× *g* at +4 °C using 5810 Eppendorf centrifuge (Eppendorf, Hamburg, Germany) to remove co-precipitated proteins and particles and ultracentrifuged for 120 min at 129,000× *g* at +4 °C (rotor SW-60, ultracentrifuge L8-70M; Beckman Coulter, Brea, CA, USA). The pellet was resuspended in TBS and filtered through a Minisart NMP Plus filter with a pore size of 0.1 μm (Sartorius, Goettingen, Germany).

The fraction obtained using ultracentrifugation was additionally purified using gel filtration on a 30 mL Ultrogel A4 column (Sigma, USA), elution rate 0.8 mL/min, equilibrated with 20 mM Tris-HCl, pH 7.5, 500 mM NaCl. The first peak enriched in exosomes was collected, dialyzed against 20 mM Tris-HCl, pH 7.5, overnight at +4 °C. Aliquots of 1 mL were frozen at –70 °C and lyophilized in FreeZone 2.5 (Labconco, Kansas, MO, USA) overnight. Lyophilized preparations were dissolved in 200 µL of Milli-Q distilled water.

### 4.3. Transmission Electron Microscopy and Nano Tracking Analysis

The preparations obtained after ultracentrifugations and gel filtration were analyzed using the negative contrast method by transmission electron microscopy (TEM). Samples were adsorbed onto a copper grid covered with a formvar film for 1 min. After incubation, we washed the grid with a drop of distilled water for 10 s and removed the excess liquid with filter paper. The contrast was performed using 0.5% uranyl acetate solution for 10 s. Grids were examined using a Jem-1400 microscope (Japan Electron Optics Laboratory, Akishima, Tokyo, Japan); images were taken using a Veleta digital camera (EMSIS, Muenster, Germany).

The size and concentration of purified horse milk exosome preparations were analyzed by nano tracking analysis under ASTM 2834–12(2018) recommendations using the Nanosight instrument LM10 HS-BF (Nanosight Ltd., Malvern, UK). A laser system with a wavelength of 405 nm and 65 mW was equipped with a susceptible EMCCD camera (Andor Luca, U.K.). The samples were diluted with TBS to the concentration of about 1.5×10^8^ particles in 1 mL; the measurements were carried out using 60 sec videos (analysis in NTA software 2.3 build 33). Each sample was measured in two technical replicates. The results of the measurements of one sample were used to calculate the mean hydrodynamic diameter and total particle concentration.

For the gold-immunolabeling, we used gold nanoparticles conjugated with Protein A (the reagent was presented courtesy of Prof. E. I. Ryabchikova). The grids with adsorbed vesicles were placed on a drop of a suspension of conjugates of gold nanoparticles and incubated for 2 h in a humid chamber at room temperature, after which they were washed for 2 min with phosphate buffer and contrasted with phosphotungstic acid for 10 s. The grids were examined using a Jem1400 as described above.

### 4.4. Affinity Chromatography on Anti-CD9- and Anti-CD63-Sepharose

Fractions of exosomes isolated from 18 horse milk preparations were merged (VESmix) and used for additional purification on CNBr-Activated Sepharose (GE Life Sciences, New York, NY, USA) with immobilized polyclonal rabbit antibodies against CD9 and CD63. The rabbits were immunized with chemically synthesized peptides NKLKTKDEPQRETLKAC and GINFNEKAIHKEGC (Biomatik, Ontario, Canada), respectively. Total IgG preparations were isolated from rabbit blood plasma using Protein G Sepharose (GE Life Sciences, New York, NY, USA) and affinity-purified on Sepharoses containing immobilized peptides. Corresponding IgGs and peptides were covalently immobilized on CNBr-Sepharose according to the manufacturer’s instructions.

Exosome preparations were applied to anti-CD9- and anti-CD63-Sepharoses equilibrated with 20 mM Tris-HCl pH 7.5; the sorbents were washed with the same buffer to zero A_280_ density (peak P1). The exosomes bound to the sorbents were eluted using buffers containing 20 mM Tris-HCl pH 7.5 and, correspondingly, 0.5 M NaCl (peak P2), 1.0 M NaCl (peak P3), and 0.1 M Gly-HCl pH 2.6 (peak P4). The fraction eluted with 0.1 M Gly-HCl pH 2.6 was immediately neutralized by 1/10 volume of 1 M Tris-HCl pH 8.8. Then, all fractions were dialyzed overnight at +4 °C in 20 mM Tris-HCl pH 7.5 and concentrated. We used these vesicle fractions for subsequent analyses.

### 4.5. Analysis of Proteins and Peptides

Horse milk exosomal proteins and peptides of 0.8–8.5 kDa were analyzed using the obtained solutions of native exosomes directly and after destruction with trifluoroacetic acid (TFA) as in [29,37]. Aliquots of 5 μL of native exosomes isolated using gel filtration and anti-CD9- and CD63-Sepharoses were mixed with 20 μL of TFA and vortexed for 30 min. The mixtures were diluted with 75 μL of water and vortexed for 10 min. We added aliquots of 100 μL acetonitrile and vortexed the mixtures for 10 min. The samples were centrifuged for 30 min at 12,000× *g* on a Minispin centrifuge (Eppendorf, Hamburg, Germany). The supernatants were carefully separated from the sediments. Peptides and proteins of extracts were analyzed using MALDI-TOF-MS/MS as described below.

To separate compounds with various molecular masses, the samples of exosomes were filtered through Amicon Ultra-4 centrifugal filters (Millipore, Darmstadt, Germany). First, we used the filters with a 30 kDa cut-off. The fractions passed through the 30 kDa membranes we applied to the 10 kDa cut-off Amicon filters. We applied the fraction with a molecular mass lower than 10 kDa to the 3 kDa cut-off filters.

For the MALDI analysis, the solutions were evaporated to the resulting 5–10 μL volumes and desalted using ZIPTip Pipette Tips C18 (Sigma, St. Louis, MO, USA) according to the manufacturer’s manual. Desalted proteins and peptides were eluted from the ZIPTips by 50% acetonitrile. The fractions with molecular masses ≤ 10 kDa were additionally treated with proteases (trypsin, chymotrypsin, and proteinase K): the proteases were added to the samples to the final concentration of 0.01 mg/mL. Reaction mixtures were incubated at +30 °C for 20 h (the control samples were incubated without proteases). As described below, 1 μL aliquots of hydrolysis products were used on MALDI.

### 4.6. MALDI Mass Spectrometry Analysis of Proteins and Peptides

The samples of horse milk exosomes were analyzed using SDS-PAGE as in [29,35]. Analysis of proteins larger than 10 kDa was performed as in [35] using MALDI-TOF mass spectrometry analysis of tryptic hydrolysates. As in [29], we analyzed peptides of horse milk exosomes after anti-CD9- and anti-CD63-Sepharose affinity chromatography. The peptides were analyzed in native vesicles eluted from anti-CD9- and anti-CD63-Sepharoses and in the samples of TFA-destructed exosomes.

We used the Autoflex speed (Bruker Daltonics, Bremen, Germany) with a nitrogen laser (337 nm) to obtain all mass spectra. The MALDI-TOF mass spectrometer was operated in the positive reflector mode (standard method RP 700–3500 Da). The spectra were analyzed in FlexControl software (version 3.4; Bruker Daltonics) [29,36,37]. We used the Peptide Calibration Standard (Bruker Daltonics, Bremen, Germany) for external spectra calibration; each spectrum was obtained by averaging 1500–5000 laser shots, 300 shots on a step, acquired at the minimum laser power necessary for ionization of the samples. The m/z spectra of small proteins and peptides were obtained.

### 4.7. Flow Cytometry Analysis

Due to their 40–100 nm diameter, exosomes cannot be analyzed by flow cytometry directly. Therefore, the exosome preparations after gel filtration and affinity chromatography on anti-CD9- and anti-CD63-Sepharoses were adsorbed to Aldehyde/Sulfate Latex Beads 4 µm (Invitrogen, Waltham, MA, USA) as in [28,36]. In short, aliquots of exosomes were stirred with latex beads in 200 μL of TBS overnight at +4 °C. Solution of 1 M glycine (1:1 volume) was added to the tubes. They were incubated for 30 min at room temperature to block unbound aldehyde/sulfate groups. The test tubes were centrifuged at 1650× *g* for 4 min using a Minispin centrifuge (Eppendorf, Hamburg, Germany) to precipitate the spheres. The precipitates were washed twice by 200 μL of 0.5% bovine serum albumin solution containing 10% fetal bovine serum.

We used the fluorescent-labeled antibodies (anti-CD81-APC, anti-CD9-FITC, and anti-CD63-Brilliant Violet A) from Biolegend (San Diego, CA, USA) to detect the CD9, CD63, and CD81 tetraspanins. Antibodies were incubated with latex beads immobilized exosomes for 30 min at +4 ° C, centrifuged for 4 min at 1650× *g*, and then we removed the supernatant. The latex beads sediments were suspended in 200 μL of TBS. Negative control samples containing latex spheres without exosomes incubated with anti-CD9, anti-CD63, and anti-CD81 fluorescent antibodies showed shallow background values. The beads were analyzed by the NovoCyte flow cytometer (ACEA Biosciences, San Diego, CA, USA). The gating strategy was the same as in [29]. The results were processed using NovoExpress 1.1.0 (Agilent, Santa Clara, CA, USA).

## 5. Conclusions

Here, we show that affinity-purified horse milk exosomes contain both CD9 and CD63 tetraspanins and a minor amount of CD81 tetraspanin, suggesting the uneven distribution of these exosome markers on the exosome surface. According to the MALDI-TOF-MS/MS data, these exosomes contain the only proteins typical for milk exosomes: CD9, CD63, CD81, lactadherin, actin, butyrophilin, lactoferrin, and xanthine dehydrogenase, and two dozen peptides with molecular masses of 0.8–8.6 kDa, which are intrinsic parts of exosomes. We cannot exclude that the peptides that we characterized in this paper may also provide some significant biological activity. We suggest that further biochemical analysis of horse milk exosomes is essential for their use as drug delivery vehicles and the use of highly purified vesicle preparations is necessary to establish the real physiological role of the milk exosomes, which are not related to the activity of co-isolating proteins.

## Figures and Tables

**Table 1:** Molecular masses of major peptides corresponding to P2 + P3 peaks of VESmix isolated by affinity chromatography on anti-CD9/CD63-Sepharoses and extracts obtained using TFA and acetonitrile.

Peptide	Molecular mass, Da *	Peptide	Molecular mass, Da
1	856.3 **^,^***	17	3471.7
2	868.8 ***	18	3476.4
3	883.4 ***	19	3805.8
4	1052.5	20	3971.7
5	1166.7 ***	21	3981.6 ***
6	1335.8 ***	22	4121.6 ***
7	1499.5 ***	23	4128.8 ***
8	1619.7 ***	24	4210.9 ***
9	1752.5 ***	25	5286.4
10	2064.5	26	5379.9 ***
11	2691.4 ***	27	6021.8 ***
12	2843.0	28	6947.8 ***
13	3006.0 ***	29	7608.5 ***
14	3013.5 ***	30	8848.5 ***
15	3327.9 ***	31	8523.8
16	3336.7 ***		

* Average values of five independent estimations are given. Depending on the analyzed peak, the standard deviation does not exceed 0.5–1.4 Da. ** Molecular masses of major reliably detected peptides corresponding to Figure 4, Figure 5 and Figure 6 are given. *** Peptides, detected in CD81-containing horse milk exosomes in [29].

## Data Availability

Not applicable.

## Supplementary Materials

The following supporting information can be downloaded at: https://www.mdpi.com/article/10.3390/ijms232416106/s1.
