# Supplementary material for: Analysis of Proteins and Peptides of Highly Purified CD9+ and CD63+ Horse Milk Exosomes Isolated by Affinity Chromatography
Source: Int J Mol Sci. 2022 Dec 17;23(24):16106. doi: 10.3390/ijms232416106 (PMC9788572; doi:10.3390/ijms232416106)
Supplement: Supplementary file 1 [file ijms-23-16106-s001.zip › ijms-2018575-supplementary.pdf]

Table S1. MALDI-TOF-MS/MS analysis of p1 horse milk exosome fraction after gel-filtration on Ultrogel A4.

Identified protein	Peptides identified[1]
CD9 tetraspanin	DVLSSFTTKPCPEAIK<br>DIQEFYK
CD63 tetraspanin	DNHTALILDR<br>GQVPDSCCVNVTQGCGTK
CD81 tetraspanin	QFYDQALQQAVVDDDANNAK<br>INLCPSGSSVLTNLFK<br>IDELFSGK
Lactadherin	TWGLNAFSWYPFYAR<br>VNMFDVPLEVQYVR
beta-Actin	SYELPDGQVITIGNER<br>GYSFTTTAER
Butyrophilin	DQLSPAVLVYQNGQER<br>GSVALLIQHVR
Lactoferrin	NLLFNDNTECLAELQGK<br>SDADLTWNSLSGK<br>YELLCPDNTR
Xanthine dehydrogenase	ITYEDLPAIITIEDAIK<br>DPPANVQLFQEVPK<br>IPAFGSIPIEFR

[1] For the identification of proteins and their molecular masses, the 2016 SwissProt program was used.
